# An Ecological-Evolutionary Investigation of Phenotypic, Genetic, and Environmental Variation and Correlations Among Reproductive Traits of Tall Goldenrod (*Solidago altissima*)

**DOI:** 10.3390/plants15010087

**Published:** 2025-12-27

**Authors:** Michael Wise, Daniel Lavy, David Carr, Warren Abrahamson

**Affiliations:** 1 Department of Biology, Bucknell University, Lewisburg, PA 17837, USA; 2 Environmental Studies Program, Roanoke College, Salem, VA 24153, USA; 3 Smilow Cancer Center, Yale Medicine, New Haven, CT 06519, USA; 4 Department of Environmental Sciences, University of Virginia, Charlottesville, VA 22902, USA

**Keywords:** fitness-related traits, genetic variation, genetic correlations, reproductive tradeoffs

## Abstract

Although fitness-related traits are expected to be under strong selection, traits related to reproduction are often quite variable within plant populations. We used data from two large greenhouse experiments to quantify phenotypic, genetic, and environmental variation, as well as genetic tradeoffs that might help explain the maintenance of within-population variation in four traits related to sexual or vegetative reproduction in tall goldenrod (*Solidago altissima*). The goldenrod population exhibited high levels of both phenotypic and genetic variation for capitulum (flower head) number and size, seed production, and rhizome growth. Significant negative genetic correlations were present between the number of capitula and size of capitula—but only at high-nutrient levels—and between seed production and rhizome growth when nutrients were more limiting. In total, negative genetic correlations may act to maintain variation in fitness-related traits in goldenrod populations—a phenomenon we suspect may be shared by other herbaceous plant species as their populations experience variation in environmental factors, such as nutrient levels, among sites or over the course of ecological succession within a site.

## 1. Introduction

A fundamental goal of ecology is to document the diversity of traits found both within and between populations of organisms. Evolutionary ecologists also seek to explain the causes of this diversity and identify factors that act to maintain intraspecific variation in traits [1,2,3]. The presence of variation in traits closely linked to fitness is particularly intriguing, as natural selection might be expected to cause the fixation of the alleles that lead to the greatest relative fitness of the individuals. A general explanation for the maintenance of variation despite selection is that not all combinations of trait values are possible within an organism [4,5,6]. In particular, a high value of one trait may come at the cost of a low value of another trait. Such tradeoffs among traits may be due to factors like competition for a limited supply of resources or negative pleiotropic effects of genes affecting traits [4,7,8]. As an explanation for variation among individuals, phenomena like these that lead to tradeoffs are key links between the fields of ecology and evolutionary biology [9,10].

Phenotypic variation among individuals is caused by both genetic diversity and differences in the environments experienced by individuals. Thus, in order to better understand the presence of variation in fitness-related traits within a population, it is first necessary to distinguish genetically controlled variation from environmentally induced variation in the traits within a population [8,10,11,12,13,14]. If there is substantial genetic variation for the traits, then negative genetic correlations between the traits may be responsible for maintaining genetic variation. Furthermore, the expression of both genetic variation in traits and genetic correlations may themselves depend on environmental conditions.

Flowering plants are well suited to such a study because of their reliance on plasticity to cope with environmental variation, as well as the variety of strategies for vegetative and sexual reproduction found throughout the angiosperms (phylum Anthophyta) [7,15]. Among angiosperm families, Asteraceae is one of the most successful in terms of number of species, geographic spread, and abundance. Its success is partly attributable to its flexibility in reproductive traits that result from its distinctive composite inflorescence (i.e., capitulum, or head). The capitulum contains a mixture of small, highly modified ray and/or disk florets). The number of capitula and the absolute and relative numbers of ray and disk florets per capitulum are species-specific characteristics, but there is often a tremendous amount of variation in these traits among individuals of the same species [16].

Within the Asteraceae, goldenrods (genus *Solidago*) take this specialization of scale to an extreme, with small capitula clustered in small groups on stalks attached to branches that are themselves attached to the rachis of a large apical panicle. A single panicle of a goldenrod plant can be composed of several thousand individual florets [17,18]. Among goldenrods, *Solidago altissima* has become a model species for the evolutionary ecology of plant ecology and plant-herbivore interactions [9,19,20,21,22,23,24,25,26,27,28,29,30]. As such, reproduction in goldenrod (both sexually through seeds and vegetatively through rhizomes) has received a considerable amount of empirical attention. The purpose of the current study is to build upon this foundation by quantifying genetic variation and environmental variance and covariance in a set of reproductive traits in a population of *S. altissima* to shed insight into factors that contribute to the maintenance of variation in fitness-related traits goldenrod, and, by extension, other early-mid successional herbaceous plants.

Specifically, we focused on four reproduction-related traits: number of capitula per plant, number of florets per capitulum (i.e., capitulum size), number of seeds produced, and rhizome mass (as an indicator of potential for vegetative propagation of new ramets). We used the results of two experiments to address three main objectives: (1) Quantify the phenotypic, genetic, and environmental variation in the traits. (2) Reveal any correlations between pairs of traits that might suggest evolutionary constraints. (3) Examine the role of environmental variation (specifically nutrient level) on the expression of genetic variation in the traits and the magnitude of genetic and environmental correlations between pairs of traits.

In addition to their fundamental relationship to potential fitness, these four reproductive traits were chosen because of the potential for tradeoffs. That is, we would expect that a plant that invests in making a greater number of capitula might be constrained to produce fewer seeds per capitulum. Likewise, a plant that invests heavily in sexual reproduction in terms of seed production might be constrained to invest in relatively lower levels of future vegetative reproduction in terms of rhizome growth.

## 2. Results

### 2.1. Variation in Reproductive Traits

**Figure 1:**
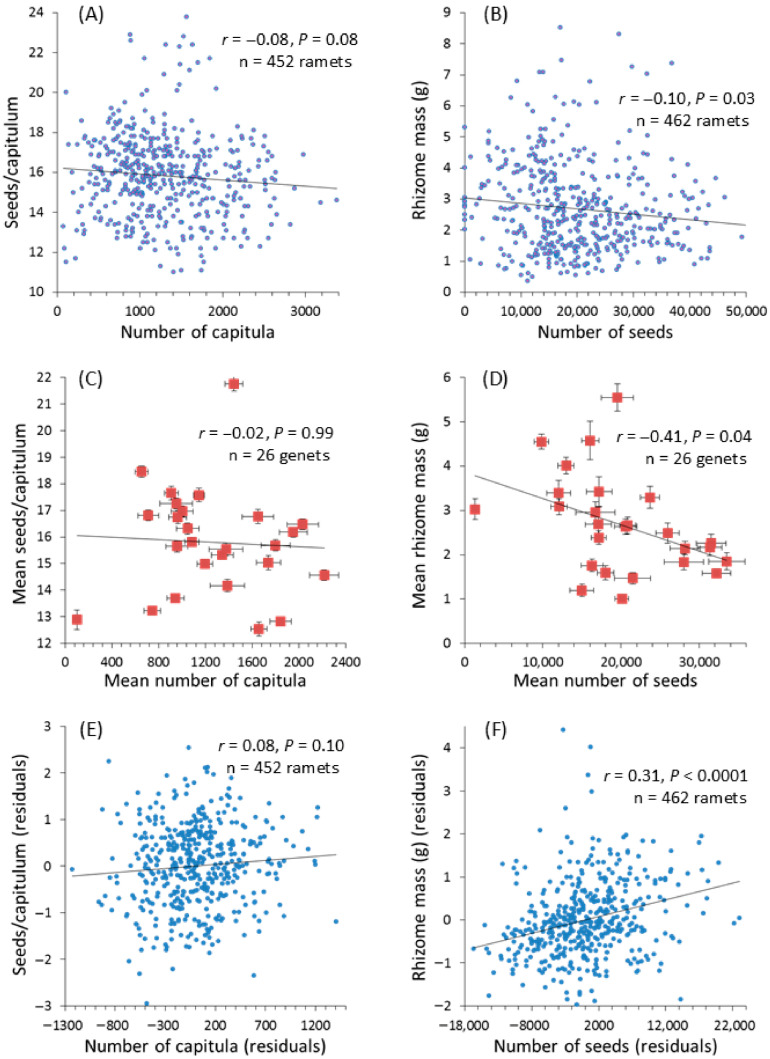
Relationships between reproductive traits in Experiment 1. The data points are untransformed values, while the correlations (*r*) and the *p*-values are from analyses that may involve transformation, as described in the text. (**A**,**B**) Phenotypic correlations, calculated as the Pearson correlations of raw phenotypic values; (**C**,**D**) Genet-mean correlations; points and bars represent genet means ± 1 SE; (**E**,**F**) Environmental correlations, which were calculated as Pearson correlations between the residuals obtained from ANOVAs of the traits with plant genet as the only explanatory variable, and thus with the influence of genet extracted from the phenotypic values.

The goldenrod plants in Experiment 1 showed a remarkable level of phenotypic variation in number of capitula and seeds, as well as in total mass of rhizomes produced (Figure 1A,B). Capitulum size (i.e., number of seeds per capitulum) showed less phenotypic variation, though the capitulum size still exceeded a twofold range (Figure 1A). Significant proportions of the variation in all four traits could be attributed to differences among genets, with broad-sense heritabilities (i.e., or clonal repeatabilities) ranging from 59 to 83% (Table 1).

### 2.2. Correlations Between Reproductive Traits

#### 2.2.1. Phenotypic Correlations

In Experiment 1, the phenotypic values of the focal traits for the ramets tended to correlate negatively with each other, though somewhat weakly. Specifically, the Pearson correlation coefficient (*r*) between the number of capitula per ramet and the capitulum size was −0.08 (*p* = 0.08, n = 452), and *r* was −0.10 (*p* = 0.03, n = 462) between the number of seeds per ramet and the rhizome mass (Figure 1A,B).

#### 2.2.2. Genetic Correlations

In Experiment 1, there was no evidence of a genetic correlation between capitula number and capitulum size (*r* = −0.02, chi-square = 0.01, *p* = 0.99; Figure 1C). In contrast, seed production was negatively genetically correlated with rhizome production (*r* = −0.41 chi-square = 4.22, *p* = 0.04; Figure 1D).

#### 2.2.3. Environmental Correlations

Unlike phenotypic and genetic correlations, environmental correlations between traits tended to be positive in direction. Although the environmental correlation was only marginally significant between capitulum number and capitulum size (*r* = 0.08 chi-square = 2.7, *p* = 0.10; Figure 1E), the environmental correlation between seed production and rhizome production was highly significant (*r* = 0.31 chi-square = 41.15, *p* < 0.0001; Figure 1F) in Experiment 1.

### 2.3. Influence of Nutrient Level

#### 2.3.1. Phenotypic Variation

Three of the four fitness-related traits expressed substantial phenotypic plasticity in response to nutrient level. Fertilized ramets produced nearly nine times as many capitula and seeds as unfertilized ramets and more than 2.7 times the mass of rhizomes (Table 2). Fertilized ramets were also an average of 1.7 times taller than unfertilized ramets (169 vs. 98 cm). In contrast, the number of seeds per capitulum was not affected by nutrient level (Table 2).

#### 2.3.2. Genetic Variation

Nutrient level did not have a consistent effect on the magnitudes of the broad-sense heritability estimates. Fertilization increased the heritabilities for the number of capitula and capitulum size by a factor of 1.24 and 1.37, respectively, but it had relatively little effect on the heritabilities for seed number or rhizome mass (Table 2). As a whole, the broad-sense heritability estimates for the subset of 15 genets in Experiment 2 were quite similar to the estimates for the set of 26 genets in Experiment 1 (Table 1), and the changes caused by fertilization were not statistically significant, based on overlaps of 95% confidence intervals.

#### 2.3.3. Genetic and Environmental Covariance

**Figure 2:**
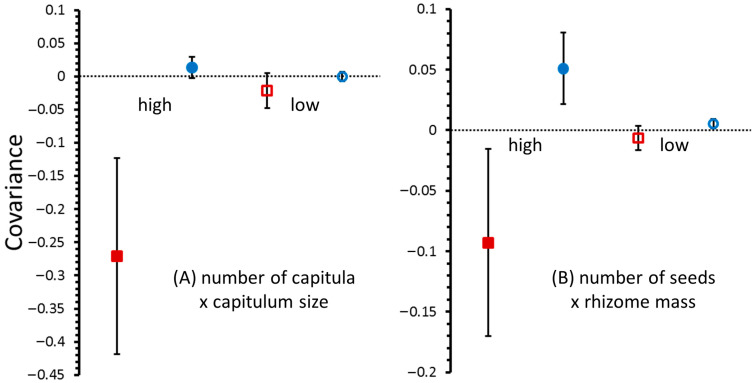
Covariances ± 1 SE between (**A**) number of capitula and capitulum size, and (**B**) number of seeds and rhizome mass. Red squares and blue circles represent genetic and environmental covariances, respectively. Filled symbols and empty symbols represent the high-nutrient and low-nutrient treatments, respectively.

Fertilization tended to increase the magnitude of the covariances between pairs of the fitness-related traits, resulting in estimates of genetic correlations that were more highly negative and environmental correlations that were slightly (but not statistically significantly) more positive than the respective correlations in the low-nutrient environment (Figure 2). Specifically, only in the fertilized treatment was the genetic correlation between the number and size of capitula statistically significant (*r* = −0.26; *p* = 0.02).

Similarly, the genetic correlation between seed production and rhizome mass was nearly twice as great in the fertilized plants (*r* = −0.41) than in the unfertilized plants (*r* = −0.21), though neither was statistically significant (*p* > 0.05).

## 3. Discussion

### 3.1. Variation in Reproductive Traits

This study documented an impressive amount of variation in key fitness-related traits among individuals derived from a single population of tall goldenrod (*Solidago altissima*). This result was especially striking considering the fact that the experiments were conducted in a controlled greenhouse environment in which variation in environmental factors was intentionally minimized. In addition, prior to the initiation of the experiments, the plants were grown from rhizome cuttings for two or three generations in controlled conditions. These propagation procedures should have reduced any carryover (i.e., maternal) effects that might have arisen from potential variation in microenvironments in the original source population. Furthermore, the ramets in the experiments were propagated from equal-sized rhizome cuttings (in terms of volume) and grown in the same commercial growing medium in equal-sized pots in the same greenhouse.

Because the heritability of a trait is the proportion of the variation within a population that is determined by genetics, the strict control of environmental conditions in these experiments would naturally be expected to lead to high values of heritability in the measured traits. In fact, the broad-sense heritabilities for the four traits in the first experiment ranged from 0.58 to 0.83. Even though the heritabilities for these traits in the experiments would almost certainly be lower if the plants were measured in their source field rather than a greenhouse, the sheer range of variation among the 26 genets tells a story of a vast amount of genetically controlled variation in fitness-related traits. Specifically, the genet-mean seed production ranged more than twenty-five-fold, from about 1300 to more than 35,500 seeds per ramet. Though not quite as variable, the genet means for number of capitula per ramet, rhizome production, and capitulum sized ranged 22-fold, more than 5-fold, and nearly two-fold, respectively.

The bottom line is that a large proportion of the variation in fitness-related traits among the individuals in the population of goldenrod from which our samples came is a result of genetic variation. Importantly, the broad-sense heritabilities calculated in this experiment represent clonal repeatabilities (which include variance due to dominance and epistasis), rather than additive genetic variation, upon which natural selection can act directly [31]. We cannot know how much of the observed genetic variation was nonadditive for any of the four traits. If even a small proportion of that variation was additive, then the plant population would have the potential to respond to natural selection for changes in the traits. Because an increase in each of the four traits would seem to be reproductively advantageous, the presence of substantial genetic variation strongly suggests that there must be some factor acting to constrain the evolutionary response to such selection.

### 3.2. Correlations Between Reproductive Traits

Negative genetic correlations between traits can slow a population’s response to natural selection directly acting to increase the means of the individual traits. This study on *Solidago altissima* revealed evidence of a tradeoff between overall sexual reproductive output and potential asexual reproduction via vegetative propagation. Specifically, the genetic correlation between seed production and rhizome growth was significantly negative (e.g., *r* = −0.41 in Experiment 1).

Both modes of reproduction are important for an herbaceous perennial plant adapted to early-mid successional fields, where disturbance may be frequent and resources may be spatially heterogeneous. Vegetative propagation is necessary for the growth of new ramets in already-established *S. altissima* populations [32,33]; therefore, genets that allocate more biomass and energy to rhizome growth may be expected to be at an advantage. At the same time, sexual reproduction (through insect-carried pollen or wind-dispersed achenes) is required for *S. altissima* to colonize newly disturbed areas. As succession proceeds within a field, the strategy of greater allocation to flower production likely becomes increasingly advantageous. Such balancing selection over time may act to maintain genetic variation in traits that promote vegetative propagation and in traits that promote sexual reproduction—as well as leading to a genetic tradeoff between traits such as rhizome production and flower production [34].

Evidence of a tradeoff in two major components of sexual reproduction was also revealed in this study. Specifically, the number of capitula a ramet produced was significantly negatively genetically correlated with the mean size of the capitula (*r* = −0.26 in the high-nutrient treatment Experiment 2). This result would appear to be an intraspecific manifestation of the classic life-history tradeoff between the number of structures and the size of structures [35]. For instance, bird species can either specialize in producing multiple small eggs or a few large eggs, but not multiple large eggs [36]. The analogy is not perfect; however, since a capitulum is more like a nest containing a “clutch” of flowers, and an inflorescence is a collection of nests. Nevertheless, biologists have expected that tradeoffs between flower (or inflorescence) size and flower (or inflorescence) should commonly occur in plant species [4,37,38,39]. Yet, negative genetic correlations between these floral traits have rarely been found in empirical investigations [4,7]. As elaborated below, one reason for this scarcity is that these correlations may only manifest under certain environmental conditions [4].

### 3.3. Influence of Nutrient Environment on Fitness-Related Traits

The addition of fertilizer increased the number of seeds and capitula and the growth of rhizomes substantially in Experiment 2. However, the differences in the means of these traits between the plants growing in high- vs. low-nutrient conditions was less than half the range displayed among the 26 genets in Experiment 1. This comparison suggests that genetic variation is likely to be responsible for a large portion of the variation in fitness-related traits in goldenrod populations—even in fields with extreme variation in nutrient levels across microhabitats. That expectation is especially true for capitulum size in *S. altissima*, which showed no phenotypic plasticity and broad-sense heritability ranging from 60% to 83% across the two experiments of this study.

Because of the lack of phenotypic plasticity in capitulum size, all of the phenotypic plasticity in seed number in response to fertilization was attributable to plasticity in the number of capitula. This result suggests that it is simple for better-nourished plants to grow larger inflorescences and add more capitula as they grow. However, the size of these capitula appears to be a much less flexible in *S. altissima*. In contrast, across species of *Solidago*, a single capitulum can contain from 0 to 24 ray florets and 2–60 disk florets (with both flower types producing seeds) [40]. This range of capitulum size across species shows that there is not a firm physical constraint as to how many flowers or seeds a goldenrod capitulum can contain. Nevertheless, capitulum size appears to be a rather canalized trait within species, and thus it can be used to distinguish among similar species of *Solidago*. The maintenance of a relatively narrow range within species suggests that there is some adaptive value to capitulum size that is idiosyncratic to each goldenrod species. Whether capitulum size is an adaptation in response to floral herbivory, pollinators, seed dispersal, or some other factor is an open and interesting question.

Agrawal and colleagues have suggested reasons why negative genetic correlations between reproductive traits may be hard to detect—and why such correlations can result even if there was not actually a selective tradeoff between the traits [4,10]. For example, tradeoffs between traits may manifest in some environmental conditions but not in others. In the current study, genetic correlations between the reproductive traits tended to be more strongly negative when the plants were growing under high-nutrient conditions—even though heritabilities of the traits were not dependent on nutrient level. In particular, if our study had not included a fertilized treatment, then we would have missed the evidence of a tradeoff between the number of capitula and size of the capitula.

On the one hand, this result runs counter to the expectation that tradeoffs are likely to be more commonly expressed in the more realistically stressful conditions of natural environments than in the relatively benign greenhouse conditions in which most experiments are performed. On the other hand, goldenrod ramets in the field are often much larger than even the fertilized ramets in our experiment—an observation that suggests that the pot-bound environments of our experiment are not necessarily benign compared to what many goldenrod plants ramets in their natural environments.

Regardless, an early-mid successional plant species like tall goldenrod is bound to experience a very wide range of ecological conditions over evolutionary time. Sometimes natural selection will favor vegetative reproduction, and other times, sexual reproduction will be favored. Sometimes there may be selection for more capitula, and other times, for more seeds per capitulum. Tradeoffs in traits caused by negative genetic correlations may slow the evolutionary response to such selection. In some environments, these tradeoffs may be expressed very strongly, and in others, hardly at all. Together, these sorts of variable ecological and evolutionary forces are likely to maintain substantial variation even in traits that are closely tied to fitness.

## 4. Materials and Methods

### 4.1. Study System

*Solidago altissima* L. (Asteraceae), or tall goldenrod, is a widespread, clonal, herbaceous weed of old-fields, roadsides, and other disturbed habitats in its native range of eastern North America, as well as in introduced ranges throughout many areas in Europe, Asia, and Africa [22,41,42,43,44]. Tall goldenrod is capable of prodigious sexual reproduction. Ramets begin flowering in late summer with a branching apical panicle containing up to several thousands of small capitula. Each capitulum of *S. altissima* bears a range of 9–15 pistillate ray flowers and 2–5 hermaphroditic disk flowers [16]. The ovaries of both types of flowers (florets) develop into single-seeded, wind-dispersed fruits called achenes. Once a genet (i.e., a genetic individual) of *S. altissima* is established by a seed in a newly disturbed site or abandoned oldfield, the genet spreads via rhizomes that send up new stems, forming clumps of genetically identical ramets. Within two years of seed recruitment, virtually all reproduction within the goldenrod population is accomplished by vegetative reproduction through rhizomes, as competition within the field on becomes too strong for additional seedlings to survive [32].

The source and propagation procedures for the goldenrod plants used in this study have been described in detail previously [17]. Briefly, rhizome samples from 26 discrete, spatially separated clumps of *S. altissima* ramets (presumed to represent 26 different genets) were excavated in spring of 2003 from a 3-ha old-field population in Union County, Pennsylvania, USA (40°57.9′ N., 76°57.3′ W). These rhizomes were grown in 27 cm diameter plastic pots in commercial growing medium (ProMix BX^TM^; Premier Horticulture Ltd., Dorval, QC, Canada). Newly sprouted rhizomes from these plants were replanted in fresh growing medium in pots each spring to purge the genets of potential carryover effects from any microhabitat differences in the source field, as well as to grow rhizomes for use in experiments that required multiple clonal replicates of the genets.

An original goal of this study was to investigate questions regarding the tolerance of *S. altissima* to feeding by a common xylem-fluid-feeding herbivore: the meadow spittlebug, *Philaenus spumarius* (L.) (Hemiptera: Aphrophoridae) [45]. The effects of spittlebugs on the traits measured in this study were minor, but not always negligible. Because spittlebug effects were not a focus of the current paper, their effects were taken into account (i.e., removed) statistically as described in the Section 4.3 below.

### 4.2. Greenhouse Experiments

This study addresses questions related to variation in four fitness-related traits using data from two greenhouse experiments conducted in 2005 and 2006. These experiments were designed to investigate a variety of questions, and design details not related to the questions of the current paper are omitted. Data from the 2005 experiment (hereafter, “Experiment 1”) are used to address questions about phenotypic, genetic, and environmental variation in fitness-related traits, as well as correlations between pairs of traits. Data from the 2006 experiment (“Experiment 2”) are used to investigate whether nutrient levels can influence relative proportions of genetic and environmental variation in the fitness-related traits, as well as the expression of correlations between the traits.

#### 4.2.1. Experiment 1

In late April of 2005, rhizomes of 26 goldenrod genets were removed from cold storage to begin plant propagation. For each genet, healthy rhizomes were cut into at least 20 equal-sized (2 cm^3^) segments, as measured by water displacement of 2 mL in a 100 mL-graduated cylinder containing 98 mL of water [46]. Rhizome segments were planted into plastic flats containing ProMix BX^TM^, and shoots (ramets) began emerging within about one week. In late May, 18 ramets from each genet were transplanted individually from the flats into 16.5 cm diameter plastic azalea pots containing ProMix BX^TM^. These 468 pots were placed in two randomized blocks on greenhouse benches at Bucknell University (representing east and west sides of the greenhouse), with one ramet per genet per each of nine spittlebug treatments per block. Early-instar spittlebug nymphs were transferred to ramets on 3 June and removed upon eclosion to adults, which concluded on 29 June. Plants were fertilized monthly using Peters Professional 15-16-17 NPK water-soluble fertilizer (J.R. Peters, Allentown, PA, USA). This fertilizer was mixed to a concentration of 3.9 mL per liter of water (i.e., 1 tablespoon per gallon), and 59 mL (1/4 cup) of the mixture was applied to each pot. In elemental terms, this rate works out 34.5 mg of N, 16.0 mg of P, and 32.5 mg of K per application.

Once a ramet finished flowering and setting seeds, its capitula were counted by hand, one inflorescence branch at a time (from 12 October to 14 November). In addition, ten capitula for each ramet were collected across different locations in the inflorescence. These capitula were dissected to count achenes and obtain a mean number of seeds per capitulum for each ramet. This mean was multiplied by the number of capitula to estimate the total number of seeds produced by each ramet. In late winter, each pot was emptied and the underground plant material was cleaned of growing medium. Roots were removed from the rhizomes, and the new rhizomes (initiated and grown in 2005) were collected into paper bags, dried to a constant mass in a drying oven at 60 °C, and weighed to the nearest mg.

During the course of the experiment, six ramets died (across five genets), leaving 462 ramets available for capitula-number, seed-number, and rhizome-mass data. Ten other ramets (including nine from one genet) did not produce any capitula; thus, only 452 ramets were available for data on capitulum size.

#### 4.2.2. Experiment 2

In April of 2006, a random subset of 15 of the 26 goldenrod genets from Experiment 1 were randomly selected for a follow-up study to investigate the effects of nutrient levels on the expression of phenotypic and genetic variation and potential correlations among the same four fitness-related traits as were examined in Experiment 1. (Spittlebugs were also a part of the experimental design, but they are not a focus of the current paper.) Goldenrod ramets were propagated from cold-stored rhizomes using the same procedures as detailed for Experiment 1. For the current study, twelve ramets per genet were grown in 16.5 cm plastic azalea pots in ProMix BX^TM^. These plants were assigned randomly to a two-way factorial design with nutrient level (n = 2) crossed by spittlebug treatment (n = 2). Each genet had three ramets per treatment combination, for a total of 180 ramets in the experiment. The high-nutrient treatment received weekly fertilization at the dosage described above for the plants in Experiment 1. The low-nutrient ramets received no fertilizer for the duration of the experiment. On 2 June, 10 field-collected, early-instar spittlebug nymphs were placed on half of the ramets. Each spittlebug was removed upon adult eclosion, which took as short as one week to as long as nearly three weeks of feeding.

Measurements of capitula number, capitulum size, seed number, and rhizome mass were taken with the same procedures as described above for Experiment 1. One of the fertilized plants died, leaving a sample size of 179 ramets for capitulum number, seed number, and rhizome mass. In addition, one of the unfertilized ramets produced no flowers, leaving a sample size of 178 ramets for the analyses involving capitulum size.

### 4.3. Statistical Analyses

#### 4.3.1. Experiment 1

In Experiment 1, bivariate analyses were used to estimate the variances and pairwise covariances of 1) the number of capitula and seeds per capitula, and 2) rhizome mass and seeds per ramet. The random effect of block had a variance component of zero in each model and was omitted from the final analyses. Genet was treated as a random factor, and the number of spittlebugs was included as a covariate to extract any variation in the traits that were caused by spittlebug feeding. To meet the distributional assumptions (e.g., minimize the skewness and kurtosis of the distribution of residuals), the number of capitula and seeds per ramet were power-transformed (raised to the power of 0.75), and the rhizome mass was natural log-transformed. The MIXED procedure in SAS (v. 9.4, SAS Institute, Cary, NC, USA) was used to estimate variance components following the two-trait design method developed by Fry [47], modified for a clonal design. Significances of variances and covariances were evaluated using log-likelihood ratio tests (1-tailed tests for variances, 2-tailed for covariances), comparing the full model to models constraining variances or covariances to 0. The residual covariances from the analyses were used to calculate the environmental correlations. Significances of the environmental covariances were also evaluated by log-likelihood ratio tests.

#### 4.3.2. Experiment 2

In Experiment 2, we again estimated the genetic and environmental variances and covariances for pairs of traits in bivariate analyses following the two-trait design method of Fry [47], modified for a clonal design. Separate analyses were run for the low- and high-nutrient environments. The data conformed to normality assumptions, but due to large differences in variances between traits, we transformed each trait to a z-score (standard normal deviate) by subtracting the mean from each trait value and dividing by the standard deviation of the trait in order to allow models to converge. Log-likelihood ratio tests were used to test whether the variances and covariances differed from zero within each environment. Differences between environments in their estimated genetic and environmental covariances were evaluated by constructing 95% confidence intervals within environments and then comparing estimates between environments.

## Figures and Tables

**Table 1:** Summary of means and variation in the four reproductive traits analyzed in Experiment 1, which included 18 ramets of each of 26 plant genets. SEM = standard error of the mean; CV = coefficient of variation = standard deviation/mean; H^2^ = percentage of variation in phenotype accounted for by genet identity (= broad-sense heritability). The means and standard errors were calculated from raw values, while the broad-sense heritability estimates and statistical inferences were based on transformed values, as described in Section 4.3.1.

Reproductive Trait	Range in Genet Means	Grand Genet Mean ± 1 SEM	CV	Broad-Sense Heritability		
				H^2^	Z-Value	*p*-Value
Number of capitula	101–2217	1264 ± 97	39	60%	3.41	0.0003
Seeds per capitulum	12.5–21.8	15.8 ± 0.4	13	83%	3.49	0.0002
Seeds per ramet	1333–33,529	19,953 ± 1519	39	58%	3.40	0.0003
Rhizome mass (g)	1.01–5.55	2.68 ± 0.22	42	59%	3.40	0.0003

**Table 2:** Ramet means ± one standard error of the mean (SEM) for four fitness-related traits, and proportion of variance explained by genetic identity (i.e., clonal repeatability or broad-sense heritability) in Experiment 2. For phenotypic means, 89 or 90 ramets (across 15 genets) were measured for each trait per nutrient treatment.

Reproductive Trait	Phenotypic Means ± SEM		Broad-Sense Heritability	
	Low Nutrients	High Nutrients	Low Nutrients	High Nutrients
Number of capitula	619 ± 38	5364 ± 165	0.58 **	0.72 **
Seeds per capitulum	17.0 ± 0.3	16.9 ± 0.2	0.60 **	0.82 **
Seeds per ramet	10,417 ± 649	89,361 ± 2579	0.59 **	0.60 **
Rhizome mass (g)	2.5 ± 0.1	6.9 ± 0.3	0.41 *	0.36 *

* *p* < 0.05, ** *p* < 0.01.

## Data Availability

The raw data collected for this study and used in the analyses are available from Dryad at the following URL: DOI https://doi.org/10.5061/dryad.f4qrfj78r.
